# NTFold: Structure-Sensing Nucleotide Attention Learning for RNA Secondary Structure Prediction

**DOI:** 10.3390/s26020688

**Published:** 2026-01-20

**Authors:** Kangjun Jin, Zhuo Zhang, Guipeng Lan, Shuai Xiao, Jiachen Yang

**Affiliations:** The School of Electrical and Information Engineering, Tianjin University, Tianjin 300072, China; jinkangjun@tju.edu.com (K.J.); lgp@tju.edu.com (G.L.); xs611@tju.edu.com (S.X.); yangjiachen@tju.edu.com (J.Y.)

**Keywords:** RNA secondary structure prediction, nucleotide attention mechanism, structure-sensing refinement

## Abstract

Determining RNA secondary structures is a fundamental challenge in computational biology and molecular sensing. Experimental techniques such as X-ray crystallography, nuclear magnetic resonance, and cryo-electron microscopy can reveal RNA structures with atomic precision, but their high cost and time consuming nature limit large-scale applications. To address this issue, we introduce the Structure-Sensing Nucleotide Attention Learning framework (NTFold), a virtual sensing framework based on deep learning for accurate RNA secondary structure prediction. NTFold integrates a Nucleotide Attention Module (NAM) to explicitly model dependencies among nucleotides, thereby capturing fine-grained sequence correlations. The resulting correlation map is subsequently refined by a Structural Refinement Module (SRM), which preserves hierarchical spatial information and enforces structural consistency. Through this two stage learning paradigm, NTFold produces high-precision contact maps that enable reliable RNA secondary structure reconstruction. Extensive experiments demonstrate that NTFold outperforms existing deep learning-based predictors, highlighting its capability to learn both local and global nucleotide interactions in an sensor inspired manner. This study provides a new direction for integrating attention driven correlation modeling with structure-sensing refinement toward efficient and scalable RNA structural sensing.

## 1. Introduction

**Figure 1:**
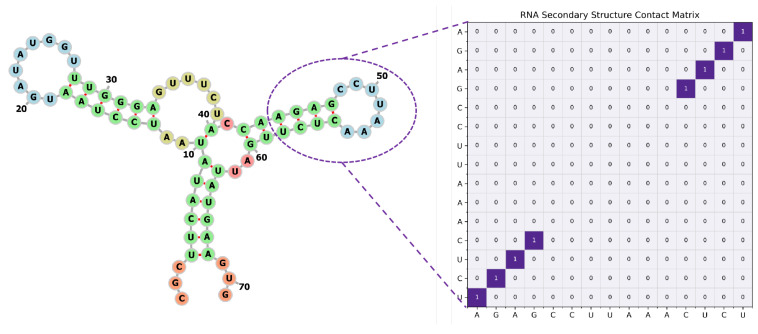
The graph and matrix representation of an RNA secondary structure.

RNA secondary structure prediction is a fundamental challenge in computational biology and is central to understanding RNA function, regulatory mechanisms, and cellular processes [1,2]. As a versatile biological macromolecule, RNA participates in a wide range of cellular activities, including gene expression regulation, catalysis, ligand sensing, and chromatin organization. Beyond its roles in modern cellular systems, RNA is also thought to have played a pioneering role in early life, where its structural versatility enabled both information storage and functional activity, supporting evolutionary plausibility under primitive conditions [3]. Unlike proteins, whose functionality is primarily encoded in the three-dimensional structure, RNA function is deeply intertwined with its secondary structure, which defines the canonical and non-canonical base-pairing patterns, as illustrated in Figure 1. These paired and unpaired regions serve as the architectural foundation for RNA tertiary folding, molecular recognition, and interactions with proteins, metabolites, and other nucleic acids [4]. Therefore, accurate secondary structure prediction is indispensable for interpreting RNA biology and for enabling RNA-design applications in synthetic biology and therapeutics.

In addition to general RNA secondary structure prediction, the modeling of pseudoknotted structures has long been recognized as a distinct and challenging problem. Early pseudoknot-oriented methods, such as HotKnots [5], extend thermodynamic folding frameworks by heuristically exploring low-energy structures that include crossing base pairs, while IPknot [6] formulates pseudoknot prediction as an integer programming problem to identify globally consistent base-pairing configurations. These approaches highlight the algorithmic complexity and diversity of strategies required to address different classes of pseudoknots. In this context, learning-based frameworks such as NTFold aim to model pseudoknotted interactions at the contact map level, providing a complementary perspective that emphasizes interaction pattern learning rather than explicit combinatorial optimization.

Despite the importance of RNA structure, determining RNA conformations experimentally remains a costly and low-throughput endeavor. Experimental techniques such as nuclear magnetic resonance (NMR) spectroscopy [7] and cryo-electron microscopy (cryo-EM) [8] can provide high-resolution structures, but they require sophisticated instrumentation, significant time investment, and often struggle with conformational heterogeneity or long and flexible RNAs. Consequently, the number of experimentally resolved RNA structures in the Protein Data Bank is orders of magnitude smaller than that of proteins, and is heavily biased toward small, stable, or highly conserved RNAs. This scarcity hinders the comprehensive characterization of structural diversity across RNA families, further emphasizing the need for robust computational prediction methods.

Computational approaches for RNA secondary structure prediction have traditionally relied on dynamic programming (DP) algorithms grounded in thermodynamic modeling. Classical energy-based methods assume that the native RNA structure corresponds to the minimum free energy (MFE) conformation and employ nearest-neighbor thermodynamic parameters to evaluate base-pairing and loop energetics [9,10]. Representative algorithms include the Zuker algorithm [11], as implemented in RNAfold [12], as well as Mfold [13] and the RNAstructure suite [14], which enumerate large numbers of candidate secondary structures and identify the MFE solution while also estimating base-pairing probabilities. Although these models have been pervasive and have served as the gold standard for decades, they suffer from several inherent limitations. First, their performance depends strongly on the completeness and accuracy of experimentally derived thermodynamic parameter sets, which remain incomplete for many complex motifs and non-canonical interactions. Second, the cubic time complexity of DP-based algorithms makes them difficult to scale to long RNA sequences and large conformational spaces. Third, structurally distinct RNA folds may exhibit near-identical free-energy values, rendering the MFE criterion ambiguous in practice. Moreover, most DP-based algorithms cannot efficiently handle pseudoknots due to prohibitive time and space complexity, leading to their routine exclusion despite biological relevance [15]. These limitations motivate alternative, data-driven approaches that infer RNA secondary structures directly from sequence patterns without explicit free-energy minimization. In contrast, NTFold adopts a data-driven learning paradigm that does not rely on predefined thermodynamic parameters or explicit energy minimization. Instead of searching for a single optimal structure, NTFold infers base-pairing patterns directly from sequence-derived interaction representations. The proposed Structural Refinement Module enforces structural consistency by reinforcing mutually exclusive base-pairing patterns and coherent helices, implicitly respecting Watson–Crick pairing rules while allowing complex interaction topologies, including pseudoknots, to be naturally represented in the predicted contact map. This paradigm shift from energy-based enumeration to pattern-driven inference highlights the novelty of NTFold and its potential advantages for scalable RNA secondary structure prediction.

Comparative sequence analysis methods provide an alternative approach by leveraging co-evolutionary information from aligned homologous sequences to identify conserved structural elements. These methods, typically formulated using stochastic context-free grammars (SCFGs) [16], exploit compensatory mutations as strong evidence for base-pairing. More recently, pipelines such as RNAcmap construct RNA contact maps by extracting co-evolutionary signals from multiple sequence alignments, providing an evolutionary constraint-driven alternative to single-sequence structure prediction [17]. While powerful in principle, their performance is fundamentally limited by the availability of sufficiently large and diverse homologous sequence families. Given that the number of well-characterized RNA families is small and unevenly distributed across organisms, comparative methods often fail when applied to novel or poorly annotated RNA sequences.

The rapid development of deep learning (DL) has transformed numerous fields, including computer vision [18,19,20], image assessment [21,22], remote sensing [23], and Internet of things [24,25]. Motivated by these successes, the application of deep learning to RNA secondary structure prediction has gained considerable momentum. Early models such as SPOT-RNA [26] integrate convolutional neural networks and bidirectional LSTMs to learn sequence-to-structure mappings. CDPFold [27] merges DL feature extraction with classical DP decoding to enhance prediction stability. Ufold [28] employs a U-Net architecture to predict contact maps by treating RNA structure prediction as an image segmentation task. E2Efold [29] and RFold [30] embed hard structural constraints directly into their unrolled optimization frameworks, thereby enforcing structural validity during decoding. MXFold2 [31] adopts multi-scale convolutional representations to capture hierarchical base-pairing patterns, while DSRNAFold [32] enhances prediction by integrating deep learning with structural context analysis. Despite these advances, many existing DL-based methods primarily emphasize feature extraction or constrained decoding, with structural validity often enforced either implicitly or at specific stages of the pipeline. In contrast, NTFold is designed to explicitly bridge data-driven interaction learning and structure-aware refinement. Rather than treating attention mechanisms solely as feature encoders, NTFold leverages attention-derived correlation maps as an intermediate structural representation and applies a dedicated Structural Refinement Module to enforce global consistency, mutual exclusivity of base-pairing, and coherent helix formation. This explicit coupling of interaction learning and structural refinement distinguishes NTFold from prior deep learning approaches and provides a more interpretable and scalable framework for RNA secondary structure prediction.

Among recent methodological advances, attention mechanisms have become a powerful paradigm for modeling interaction dependencies and have achieved remarkable success in computer vision [33], medical image analysis [34,35], and biomedical applications [34,35]. Motivated by their strong capability in capturing long-range relational patterns, several RNA secondary structure prediction models have incorporated attention mechanisms to enhance the modeling of base-pairing interactions. For example, ATTfold explicitly integrates attention layers with structural constraints to model long-range dependencies, including pseudoknotted interactions [36], while other approaches embed attention within end-to-end prediction pipelines [29]. However, most existing attention-based approaches treat nucleotide interactions implicitly within transformer-style architectures, where attention primarily serves as a feature reweighting mechanism rather than an explicit structural representation. As a result, nucleotide–nucleotide dependencies are not directly formulated or refined as interpretable interaction maps, which may obscure fine-grained relational cues embedded in primary sequences and limit the extraction of biologically meaningful correlation patterns. In contrast, NTFold elevates attention-derived correlation maps to a central structural representation, which are subsequently refined to enforce global consistency and mutually exclusive base-pairing patterns, thereby providing a clear methodological departure from prior attention-based RNA secondary structure predictors.

In contrast, this work takes a more biologically grounded perspective by designing the model as a functional analogue of a structural sensor that directly perceives and interprets nucleotide relationships. To this end, a Nucleotide Attention Module (NAM) is introduced to explicitly construct a high-resolution interaction map by modeling how each nucleotide responds to and influences others within the sequence. This produces an interpretable, attention-driven correlation representation that reflects the underlying biochemical dependency patterns. Building upon this representation, we further develop a Structural Refinement Module (SRM) to refine the initial interaction map into a biologically coherent contact matrix, leveraging hierarchical structural features. Together, these components allow the model to function as a biologically inspired sensing system that detects, integrates, and interprets nucleotide interactions, ultimately enabling accurate RNA secondary structure prediction. Our contributions are as follows:

(1) We introduce NTFold, a virtual sensing framework that enables efficient and scalable RNA secondary structure prediction, providing a computational alternative to costly and low-throughput experimental structure determination.

(2) We design a Nucleotide Attention Module that explicitly captures nucleotide dependencies, enabling fine-grained sensing of sequence-level correlations.

(3) We develop a structure refinement module that enhances spatial consistency and improves the accuracy of the predicted contact map, enabling more reliable RNA secondary structure reconstruction.

## 2. Related Work

### 2.1. Thermodynamic Models

Traditional methods for RNA secondary structure prediction are primarily based on thermodynamic principles, with the core idea of finding the structure with the Minimum Free Energy (MFE). The pioneering work of Zuker and Nussinov laid the foundation for this field, proposing dynamic programming algorithms that can find the optimal non-pseudoknotted structure in polynomial time [11,37]. Mfold [13] is a widely used web-based RNA and DNA secondary structure prediction tool that employs thermodynamic free-energy minimization to generate candidate folding structures and related stability analyses from single-stranded nucleic acid sequences. Tools such as the ViennaRNA package (RNAfold) [12] and RNAstructure [14] are widely used, leveraging experimentally determined thermodynamic parameters to evaluate and predict structural stability. However, these methods face two major challenges: first, their computational complexity (typically $O(N^{3})$) becomes prohibitive for long RNA sequences; second, they struggle to handle complex pseudoknotted structures, with complexity increasing to $O(N^{6})$ or higher, limiting their application in genome-scale analysis.

### 2.2. Hybrid Methods

Hybrid methods combine the advantages of multiple strategies to overcome the limitations of single methods. RNAalifold, an early hybrid method in the ViennaRNA package, combines energy-based and comparative methods. It calculates the minimum energy structure of a set of aligned input sequences by modifying the scoring scheme of the dynamic programming algorithm in traditional thermodynamic methods [38]. CentroidFold is based on the γ-centroid estimator and supports multiple probabilistic models, providing more accurate predictions [39]. MXFold integrates thermodynamic information with structured support vector machines (SSVMs) and predicts the optimal secondary structure through a Zuker-style dynamic programming algorithm, improving the prediction accuracy and robustness [40]. MXFold2 further improves on this basis. It replaces SSVMs with deep neural networks, combines CNN and BiLSTM layers to learn folding parameters, and calculates the final score through a dynamic programming algorithm, showing excellent performance on multiple test datasets [31].

### 2.3. Deep Learning-Based Methods

Deep learning-based approaches have gained significant traction in RNA secondary structure prediction due to their ability to model complex relationships between RNA sequences and their structural features. SPOT-RNA [26] combines ResNet and bidirectional LSTM architectures, leveraging transfer learning to predict all base pairs, including non-canonical and pseudoknotted ones. CDPFold [27] integrates convolutional neural networks (CNNs) with dynamic programming to enhance prediction accuracy. E2Efold [29] is an end-to-end deep learning model that has made significant progress in pseudoknot prediction. MXFold2 [31] combines deep learning with thermodynamic models, employing a multi-scale convolutional neural network to handle long sequences and capture complex base-pairing patterns [31]. Ufold [28] employs a U-Net architecture to represent RNA sequences as “images” for structural inference. RFold [30] proposes a framework that leverages solutions to the K-Rooks problem to guide RNA secondary structure prediction, ensuring structural constraints are met during inference. DSRNAFold [32] combines deep learning techniques with structural context analysis to improve RNA secondary structure prediction. These methods collectively demonstrate the potential of deep learning in advancing RNA structural modeling.

## 3. Method

### 3.1. Overview of the Proposed Framework

**Figure 2:**
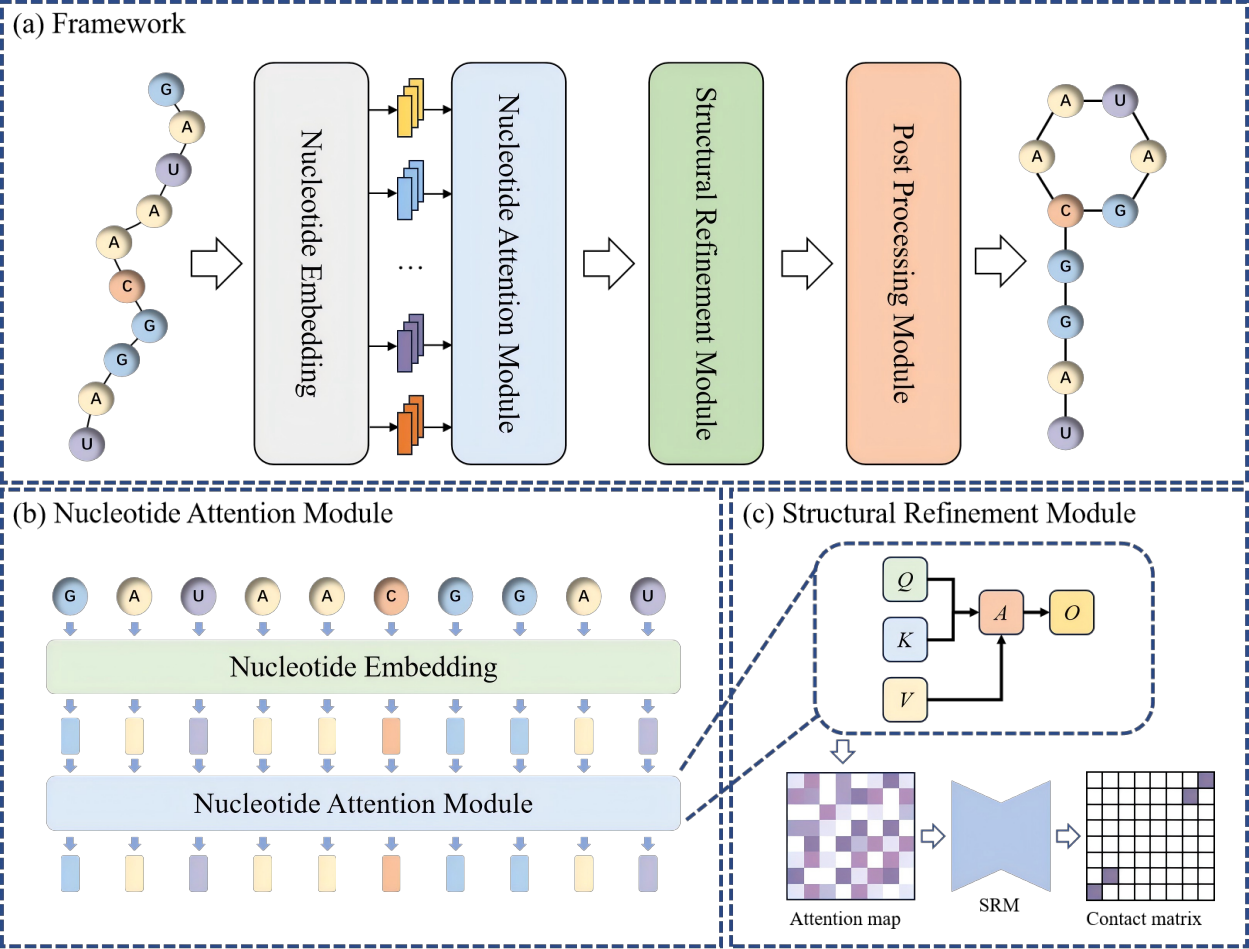
The overall framework of NTFold.

In this study, we propose an efficient deep learning framework for RNA secondary structure prediction, which directly maps nucleotide sequences to base-pair contact maps. The overall pipeline consists of three key components: (1) A NAM for constructing pairwise correlation maps; (2) A SRM for enhancing structural consistency; (3) A post-processing module for generating the final contact map. The complete workflow is illustrated in Figure 2.

Given an input RNA sequence, each nucleotide is first encoded as a learnable embedding vector to capture local sequence context. The embedding sequence is then processed by a multi-head self-attention module to model global dependencies and infer intrinsic pairwise interaction tendencies. Aggregating the attention weights yields an initial correlation map, which serves as an initial interaction map.

Despite capturing global interactions, the raw attention map often contains noise and weak long-range signals. To address these issues, we employ a structural refinement module, which extracts multi-scale features and reconstructs the interaction map. This process strengthens canonical and non-canonical pairing patterns and suppresses spurious correlations, resulting in a refined interaction map that better reflects RNA structural principles.

Finally, the contact map generation module applies structural constraints to produce a binary base-pair contact map. Overall, our framework integrates global dependency modeling via attention with spatial refinement through structural refinement module, enabling accurate and interpretable RNA secondary structure prediction without reliance on manual features or thermodynamic models.

### 3.2. Nucleotide Embedding

Given an RNA sequence $x=\{x_{1},x_{2},\ldots ,x_{L}\}$ of length *L*, we first convert each nucleotide into a learnable embedding vector. This step transforms discrete categorical symbols into a continuous representation space that captures their biochemical properties and contextual significance. Formally, we define a trainable embedding matrix $E\in R^{|V|\times d}$, where V={A,U,G,C,N} denotes the nucleotide embedding and *d* is the feature dimension. The initial embedding is computed by

(1) $$e_{i}=E\left(x_{i}\right),\;i=1,2,\ldots ,L.$$

To incorporate positional dependency, which is essential for capturing non-local interactions and structural motifs, we add a deterministic positional encoding term $p_{i}$ to each embedding:

(2) $$z_{i}=e_{i}+p_{i},$$

where $p_{i}$ encodes the relative order along the RNA chain, enabling the model to distinguish nucleotides with identical identities but different positions. The resulting embedding matrix is

(3) $$Z=\left[z_{1};z_{2};\ldots ;z_{L}\right]\in R^{L\times d}.$$

This embedding representation serves as the input feature backbone for downstream attention inference and structural refinement. It ensures that both nucleotide identity and positional context are preserved, providing a biologically meaningful representation for subsequent interaction modeling.

### 3.3. Nucleotide Attention Module

To explicitly model long-range nucleotide dependencies and infer potential pairing tendencies, we employ a multi-head self-attention mechanism that transforms the embedding features into an interaction-aware representation. Given the embedding matrix

$$Z\in R^{L\times d},$$

we compute, for each attention head *h*, a query, key, and value triplet through learned linear projections:

(4) $$Q_{h}=ZW_{h}^{Q},\;K_{h}=ZW_{h}^{K},\;V_{h}=ZW_{h}^{V},$$

where $W_{h}^{Q},W_{h}^{K},W_{h}^{V}\in R^{d\times d_{h}}$ are trainable matrices and $d_{h}$ denotes the representation dimension per head.

The interaction strengths between nucleotide positions *i* and *j* are quantified through scaled dot-product attention:

(5) $$A_{h}=\mathrm{Softmax}\left(\frac{Q_{h}K_{h}^{⊤}}{\sqrt{d_{h}}}\right),$$

where $A_{h}\left(i,j\right)$ reflects how strongly nucleotide $x_{i}$ attends to nucleotide $x_{j}$. To obtain a unified correlation estimate, we average all attention heads:

(6) $$C=\frac{1}{H}\sum_{h=1}^{H}A_{h},\;C\in R^{L\times L}.$$

The resulting matrix C provides a coarse estimation of intrinsic nucleotide interaction likelihoods, capturing canonical pairing patterns (e.g., A–U, C–G) as well as non-canonical structural relationships over long distances.

Unlike sequential convolution, attention enables direct global receptive fields without increasing model depth, allowing distal nucleotides involved in stems, pseudoknots, or long hairpins to influence each other immediately. Thus, C forms the initial structural correlation map that bridges sequence encoding with spatial structural inference, serving as the input to the refinement module for spatially coherent reconstruction.

### 3.4. Structural Refinement Module

The correlation map $C\in R^{L\times L}$ obtained from the Nucleotide Attention Module provides a coarse estimate of base-pairing propensities. However, it often contains noise, fragmented helices, and weak long-range signals. To address these issues, we introduce a Structural Refinement Module, which refines C into a more spatially coherent interaction map $C^{\mathrm{refined}}$.

The refinement module is implemented as a U-Net style encoder–decoder network. The encoder progressively extracts multi-scale features:

(7) $$F_{k}={\mathrm{ConvBlock}}_{k}\left(\mathrm{Down}(F_{k-1})\right),\;F_{0}=C,$$

where k=1,…,n, Down(·) denotes a downsampling operation, and $F_{k}$ captures structural features at different nucleotide scales, with shallow layers focusing on local motifs and loop-level structures within approximately 30 nucleotides, and deeper layers modeling global structural context characterized by long-range dependencies spanning hundreds of nucleotides.

The decoder restores spatial resolution while integrating skip connections from the encoder:

(8) $$G_{k}={\mathrm{ConvBlock}}_{k}^{\prime }\left(\mathrm{Up}\left(G_{k-1}\right)\|F_{n-k}\right),$$

where Up(·) denotes upsampling, ‖ represents feature concatenation, and *n* is the total number of encoder/decoder layers. Canonical pairing patterns are strengthened due to their consistently high attention scores and spatial continuity in the correlation map, whereas non-canonical pairs are retained when they form stable, context-supported interaction patterns across adjacent positions. The refinement process favors such recurrent structural signals while filtering out noise-like, unsupported interactions.

Finally, the refined correlation map is obtained as

(9) $$C^{\mathrm{refined}}=\mathrm{SRM}\left(C\right),\;C^{\mathrm{refined}}\in R^{L\times L}.$$

The Structural Refinement Module thus produces a high-quality interaction map that better respects RNA structural principles, serving as the input to the post-processing module for binary contact map generation. By integrating multi-scale local and global features, this module strengthens helix continuity, improves structural consistency, and enhances the reliability of downstream RNA secondary structure reconstruction.

### 3.5. Post-Processing Module

The refined correlation map $C^{\mathrm{refined}}\in R^{L\times L}$ produced by the Structural Refinement Module encodes base-pairing propensities with reduced noise and improved helix continuity. To convert this map into a biologically valid RNA secondary structure, we apply a post-processing step that enforces structural constraints and generates the final binary contact map $M\in {\left\{0,1\right\}}^{L\times L}$.

We first impose a sparsity-aware thresholding on $C^{\mathrm{refined}}$ using a smooth approximation of the sign function:

(10) $$\tilde{C}=\mathrm{soft\_sign}\left(C^{\mathrm{refined}}-s\right)⊙C^{\mathrm{refined}},$$

where *s* is a predefined threshold and ⊙ denotes element-wise multiplication. This step suppresses weak interactions while retaining strong base-pair signals.

Next, we enforce Lagrangian constraints to satisfy RNA pairing rules. Let A denote the contact probabilities after constraint optimization. The update rule for A is

(11) $$G=A⊙\left(\lambda \cdot \mathrm{soft\_sign}(\sum_{j}A_{ij}-1)\right)-\frac{1}{2}A,$$

(12) A←A−ηG,

(13) $$\lambda \leftarrow \lambda +\eta_{\lambda }\cdot \mathrm{ReLU}\left(\sum_{j}A_{ij}-1\right),$$

where λ is a Lagrange multiplier and η and $\eta_{\lambda }$ are learning rates for the minimization and maximization steps, respectively, and the sum is taken over each nucleotide to ensure that each pairs with at most one partner.

Finally, we generate the binary contact map M by applying the following structural constraints: (1) Symmetry: $M=M^{⊤}$; (2) Pairing exclusivity: Each nucleotide pairs with at most one partner; (3) Local masks: Prohibit impossible pairings due to minimum loop lengths or steric constraints.

The resulting map M represents a valid RNA secondary structure, ready for downstream analysis or visualization. This post-processing module integrates constraint optimization with biologically motivated rules, ensuring that the final predictions are both accurate and structurally plausible.

## 4. Experiments

### 4.1. Dataset Selection and Analysis

To comprehensively evaluate the proposed model, we adopt three widely used RNA secondary structure datasets as benchmarks, including RNAStrAlign [41], bpRNA-1m [42], and ArchiveII [43]. These datasets cover diverse RNA families and a broad range of sequence lengths, providing a solid foundation for assessing model accuracy, robustness, and generalization performance.

**RNAStrAlign:** RNAStrAlign [41] contains 30,451 RNA secondary structures spanning eight RNA families. After applying quality control and sequence-length filtering, we obtain a refined training set containing 20,862 high-quality RNA samples. The distribution of sequence lengths and sample counts across families is summarized in Table 1. This analysis also reveals distinct sparsity characteristics among different RNA families, which is crucial for evaluating the model’s capability in handling variable sparsity patterns.

**ArchiveII:** ArchiveII [43] contains 3975 experimentally validated RNA secondary structures and has been widely adopted as a benchmark for RNA secondary structure prediction. In this work, we use ArchiveII exclusively for evaluating the model trained on RNAStrAlign, thereby assessing its generalization ability across datasets.

**bpRNA-1m:** For the bpRNA-1m dataset [42], which contains 102,318 sequences from 2588 families, we followed the pre-processing protocol of MXfold2 [31] by using CD-HIT to remove redundant sequences, and then randomly divided the remaining data into training and testing subsets, denoted as TR0 and TS0.

### 4.2. Evaluation

We evaluate model performance using precision, recall, and F1 score. Precision measures the proportion of correctly predicted base pairs among all predicted base pairs, while recall quantifies the proportion of true base pairs that are successfully recovered. The F1 score is defined as the harmonic mean of precision and recall, providing a balanced evaluation of base-pair prediction accuracy that accounts for both false positives and false negatives. These metrics are computed as follows:

(14) $$\mathrm{Precision}=\frac{TP}{TP+FP},$$

(15) $$\mathrm{Recall}=\frac{TP}{TP+FN},$$

(16) $$F1=\frac{2\times \mathrm{Precision}\times \mathrm{Recall}}{\mathrm{Precision}+\mathrm{Recall}},$$

where TP denotes the number of correctly predicted base pairs, FP represents the number of falsely predicted base pairs, and FN indicates the number of missed true base pairs.

### 4.3. Standard RNA Secondary Structure Prediction

Table 2 presents the performance comparison of different RNA secondary structure prediction methods on the RNAStrAlign test set. Traditional thermodynamic approaches such as Mfold, RNAfold, and RNAstructure achieve relatively low performance, with F1 scores ranging from 0.42 to 0.55. With the incorporation of statistical learning and efficient inference strategies, methods like CONTRAfold, LinearFold, and CDPfold show moderate improvements, reaching F1 values around 0.61–0.63. Deep learning-based approaches demonstrate a substantial performance gain, where UFold achieves an F1 score of 0.915, indicating stronger capability in detecting true base-pairing patterns. In comparison, our proposed model achieves the best overall performance, obtaining 0.979 in Precision, 0.976 in Recall, and 0.977 in F1 score. This represents an improvement of approximately 6.2% over UFold in terms of F1, highlighting the superiority of our method in accurately capturing base-pair dependencies and delivering more reliable structural predictions.

### 4.4. Evaluating Model Generalization

Table 3 reports the performance of various RNA secondary structure prediction approaches on the ArchiveII dataset. Traditional thermodynamic models such as Mfold, RNAfold, RNAstructure, and RNAsoft achieve moderate performance, with F1 scores generally ranging from 0.60 to 0.65. Methods incorporating probabilistic modeling or structural constraints, including CONTRAfold, LinearFold, and Contextfold, yield notable gains, with Contextfold reaching an F1 score of 0.842, demonstrating the advantage of more expressive modeling strategies. Deep learning-based approaches exhibit substantial improvements over classical methods. Early neural frameworks, such as E2Efold and SPOT-RNA, already show clear performance advantages, while more recent architectures including MXfold2, RTfold, UFold, DSRNAFold, and Sincfold achieve further improvements in both precision and recall. Notably, Sincfold attains the highest recall 0.953 and an F1 score of 0.923, indicating strong capability in capturing true base-pairing interactions. Our proposed method achieves the best overall performance, obtaining the highest precision of 0.941 and the highest F1 score of 0.931, while maintaining competitive recall of 0.933. This demonstrates that our model yields more accurate base-pair predictions with fewer false positives, while preserving strong sensitivity to true interactions.

Recent studies have shown that the generalization ability of deep learning-based RNA secondary structure predictors is strongly influenced by sequence similarity and RNA family distribution between training and test sets. In particular, Qiu et al. demonstrated that apparent performance gains may diminish under distribution shifts when homologous or structurally related sequences are excluded, highlighting sequence similarity as a key determinant of generalizability [47]. In this context, the dataset splits used in our evaluation are designed to mitigate information leakage across RNA families, providing a more stringent assessment of out-of-distribution performance. More broadly, as summarized in the review by Zhao et al., machine learning-based RNA secondary structure predictors can be categorized by their modeling assumptions, input representations, and decoding strategies, each exhibiting distinct generalization behaviors across RNA families [48]. Our results should therefore be interpreted within this framework, where improved performance reflects both architectural design and the ability to capture transferable structural patterns beyond sequence-level similarity. Overall, these results confirm that our approach establishes a new state-of-the-art on the ArchiveII benchmark, outperforming classical, probabilistic, and deep learning-based predictors, thereby validating the effectiveness and strong generalization capability of our proposed framework.

**Table 3:** Results on ArchiveII dataset. The best results are highlighted in bold, and the second-best results are underlined.

Method	Precision	Recall	F1
Mfold [13]	0.668	0.590	0.621
CDPfold [27]	0.557	0.535	0.545
RNAfold [12]	0.663	0.613	0.631
RNAstructure [44]	0.664	0.606	0.628
CONTRAfold [45]	0.696	0.651	0.665
LinearFold [46]	0.724	0.605	0.647
RNAsoft [49]	0.665	0.694	0.622
E2Efold [29]	0.734	0.660	0.686
SPOT-RNA [26]	0.743	0.726	0.711
MXfold2 [31]	0.788	0.760	0.768
Contextfold [50]	0.873	0.821	0.842
RTfold [51]	0.891	0.789	0.814
UFold [28]	0.887	0.928	0.905
DSRNAFold [32]	0.896	0.927	0.907
Sincfold [52]	0.908	**0.953**	0.923
Ours	**0.941**	0.933	**0.931**

### 4.5. Large-Scale Benchmark Evaluation

Table 4 reports the performance of mainstream RNA secondary structure prediction approaches on the bpRNA-TS0 test set, which is constructed under the TR0–TS0 separation strategy to evaluate true generalization to unseen structural families. Traditional thermodynamic models, such as RNAfold, Mfold, and RNAstructure, yield moderate performance with F1 scores around 0.53–0.54. Methods incorporating machine learning priors, such as CONTRAfold and Contextfold, achieve slightly higher accuracy, though improvements remain limited. Among early neural models, SPOT-RNA achieves the highest recall of 0.693, indicating strong sensitivity to true base-pairing signals, yet its precision remains moderate, reflecting a higher false positive rate. Recent data-driven approaches demonstrate further enhancements. DSRNAFold shows a notable improvement in precision of 0.641 and achieves an F1 score of 0.627, reflecting better structural consistency. Sincfold obtains the highest precision of 0.702, but its lower recall reduces its ability to fully recover true interactions, limiting overall performance. Our method achieves the highest F1 score of 0.645, establishing the best balance between precision and recall among all evaluated models. Compared to MXfold2—the baseline corresponding to this dataset split—our method improves precision and recall by 11.3% and 6.7%, respectively. This indicates that our approach not only reduces structural false positives but also improves the recovery of true base pairs. Overall, the results on bpRNA-TS0 confirm that our method demonstrates superior robustness and generalization under distribution shifts, effectively handling previously unseen RNA structural patterns.

### 4.6. Pseudoknot Prediction

Table 5 presents pseudoknot prediction performance on the RNAStrAlign benchmark. Classical thermodynamic approaches such as RNAstructure achieve reasonable overall accuracy F1 of 0.769, yet their recall is limited, indicating challenges in capturing complex base-pairing patterns. Reinforced by deep learning, SPOT-RNA substantially improves recall by 0.978, but at the expense of precision, reflecting over-prediction tendencies. More recent end-to-end neural architectures, including E2Efold and UFold, further boost both metrics, with UFold achieving strong balance F1 of 0.976. Our method achieves the best results overall, obtaining the highest precision of 0.980 and the highest F1 score of 0.977, demonstrating superior capability in accurately identifying pseudoknots while minimizing false positives. These results highlight that our approach offers more reliable structural modeling, particularly for complex RNA folding patterns that are known to be challenging for existing predictors.

### 4.7. Ablation Experiment

The results in Table 6 clearly highlight the effectiveness of both the Nucleotide Attention Module and the Refinement Block within our model architecture. When either component is removed, the performance consistently drops across all three datasets, demonstrating their complementary contributions.

First, removing the Nucleotide Attention Module causes the most noticeable degradation. For example, on the RNAStralign dataset, the F1 score decreases from 0.977 to 0.927, showing that attention significantly enhances base-pair interaction modeling by explicitly capturing nucleotide-wise contextual dependencies. A similar trend is observed on ArchiveII and bpRNA-TS0, where F1 drops from 0.931 to 0.911 and from 0.645 to 0.623, respectively. This indicates that nucleotide-level attention is crucial for learning more accurate long-range pairing relations and improving prediction robustness across diverse RNA families.

Second, removing the Refinement Block also results in performance decline, though to a lesser extent compared with removing attention. For RNAStralign, the F1 decreases from 0.977 to 0.953, and similar performance gaps appear on ArchiveII and bpRNA-TS0. These results confirm that the Refinement Block further improves the initially predicted interaction map by suppressing noise and enhancing structural consistency, leading to more precise base-pair confidence estimation.

Overall, the full model consistently achieves the best results under all evaluation metrics. The complementary effects of Nucleotide Attention in capturing fine-grained dependencies and the Refinement Block in boosting structural refinement collectively drive the strong performance of our approach.

### 4.8. Inference Time Comparison

The inference time comparison in Table 7 demonstrates that our proposed method achieves highly efficient RNA structure prediction while maintaining strong accuracy. For short sequences of 128 nucleotides, our model completes inference in only 0.032 s per sample, making it the fastest among all existing GPU-based predictors. Compared with widely used deep learning methods such as MXfold2 0.204s, SPOT-RNA 0.847s, and SincFold 0.267s, our approach reduces latency by a significant margin, indicating a more streamlined computation pipeline. When handling longer RNA sequences 512 nt, our method retains the same efficiency advantage. The inference time increases only slightly to 0.154 s per sequence, which is still substantially lower than MXfold2 3.117s, SPOT-RNA 1.877s, and DSRNAFold 0.756s. Even relative to UFold, a known efficient model, our method achieves approximately twice the speed improvement. This stable scalability across sequence lengths suggests that our design avoids excessive computation growth when modeling long-range dependencies. It is worth noting that, beyond learning-based predictors, computational efficiency in RNA folding has also been addressed through algorithmic approximations within the thermodynamic framework. Representative methods such as LinearPartition approximate ensemble partition functions in linear time, offering an alternative notion of tractability by trading exactness for scalability [53]. While such approaches do not directly compete with single-sequence structure predictors or contact map-based models, they provide an important complementary baseline for understanding speed–accuracy trade-offs in RNA structure analysis. In summary, the results show that our model simultaneously achieves high prediction accuracy and fast inference speed. The reduced per sequence runtime and favorable scalability make it suitable for large-scale RNA datasets and real-time downstream biological applications.

## 5. Discussion

The experimental results indicate that NTFold not only improves predictive accuracy but also recovers biologically meaningful RNA secondary structure patterns. Analysis of the predicted contact maps shows that the model consistently captures canonical base-pairing signals and forms continuous stem regions, while loop boundaries are clearly distinguished by attenuated interaction strengths, reflecting known RNA folding rules. Structural comparisons across RNAs from the same families further suggest that conserved stem–loop organizations are preserved even under sequence variation, indicating that the model learns family-level structural regularities rather than relying solely on local sequence features. Compared with previous deep learning models such as convolutional or U-Net-based predictors, the explicit nucleotide attention mechanism in NTFold provides a clearer representation of long-range dependencies and yields interpretable correlation maps that reveal nucleotide interaction tendencies. The ablation studies support this interpretation: removing the attention module weakens long-range base-pair recovery, while excluding the refinement module leads to fragmented helices and increased spurious contacts, confirming their complementary roles in capturing global dependencies and enforcing structural coherence. Although challenges remain in modeling complex tertiary interactions and multiple metastable conformations, these results demonstrate that NTFold aligns well with established RNA structural principles and offers both improved performance and mechanistic interpretability.

## 6. Conclusions

In this work, we present a data-driven framework for RNA secondary structure prediction designed for molecular sensing and diagnostic applications. The model combines nucleotide-level attention, spatial refinement, and constraint-driven decoding to generate accurate and biologically consistent RNA contact maps directly from sequences, without relying on thermodynamic folding algorithms. By capturing global interaction tendencies through attention-derived correlation maps and enhancing structural continuity via refinement, the framework improves the detectability of motifs such as stems and loops that are critical for RNA-based sensors. Constraint-driven decoding ensures biologically plausible structures, making the predictions applicable to molecular engineering tasks. The method demonstrates competitive accuracy, interpretability, computational efficiency, and modularity, supporting integration into pipelines for aptamer design, CRISPR guide optimization, and analysis of target-induced structural changes. Overall, this framework bridges deep learning-based structural modeling with practical RNA sensing needs, offering both methodological innovations and applied value for biosensing research.

## Figures and Tables

**Table 1:** Analysis on RNAStralign training set.

Type	Length	Samples
16SrRNA	54∼600	1226
5SrRNA	104∼132	9133
tRNA	59∼95	7401
grp1	163∼600	1683
SRP	30∼553	493
tmRNA	102∼437	514
RNaseP	189∼486	379
telomerase	382∼559	33

**Table 2:** Results on RNAStralign test set. The best results are highlighted in bold, and the second-best results are underlined.

Method	Precision	Recall	F1
Mfold [13]	0.450	0.398	0.420
RNAfold [12]	0.516	0.568	0.540
RNAstructure [44]	0.537	0.568	0.550
CONTRAfold [45]	0.608	0.663	0.633
LinearFold [46]	0.620	0.606	0.609
CDPfold [27]	0.633	0.597	0.614
E2Efold [29]	0.866	0.788	0.821
UFold [28]	0.905	0.927	0.915
Ours	**0.979**	**0.976**	**0.977**

**Table 4:** Results on bpRNA-TS0 set. The best results are highlighted in bold, and the second-best results are underlined.

Method	Precision	Recall	F1
E2Efold [29]	0.140	0.129	0.130
RNAstructure [44]	0.494	0.622	0.533
RNAsoft [49]	0.497	0.626	0.535
RNAfold [12]	0.494	0.631	0.536
Mfold [13]	0.501	0.627	0.538
Contextfold [50]	0.529	0.607	0.546
LinearFold [46]	0.561	0.581	0.550
MXfold2 [31]	0.519	0.646	0.558
CONTRAfold [45]	0.528	0.655	0.567
SPOT-RNA [26]	0.594	**0.693**	0.619
UFold [28]	0.521	0.588	0.553
DSRNAFold [32]	0.641	0.649	0.627
Sincfold [52]	**0.702**	0.604	0.624
Ours	0.632	0.689	**0.645**

**Table 5:** Pseudoknot prediction results on RNAStralign dataset. The best results are highlighted in bold, and the second-best results are underlined.

Method	Precision	Recall	F1
RNAstructure [44]	0.778	0.761	0.769
SPOT-RNA [26]	0.677	0.978	0.800
E2Efold [29]	0.844	0.990	0.911
UFold [28]	0.962	**0.990**	0.976
Ours	**0.980**	0.976	**0.977**

**Table 6:** Ablation experiment results of RNAStralign, ArchiveII, and bpRNA-TS0 datasets. The best results are highlighted in bold. The w/o denotes the variant without the corresponding module.

RNAStralign Dataset			
Method	Precision	Recall	F1
NTFold	**0.979**	**0.976**	**0.977**
w/o Nucleotide Attention	0.926	0.938	0.927
w/o Refinement Block	0.951	0.961	0.953
**ArchiveII Dataset**			
Method	Precision	Recall	F1
NTFold	**0.941**	**0.933**	**0.931**
w/o Nucleotide Attention	0.912	0.906	0.911
w/o Refinement Block	0.923	0.918	0.919
**bpRNA-TS0 Dataset**			
Method	Precision	Recall	F1
NTFold	**0.632**	**0.689**	**0.645**
w/o Nucleotide Attention	0.601	0.657	0.623
w/o Refinement Block	0.622	0.667	0.631

**Table 7:** Inference time on RNAStralign test set.

Method	Time/Seq (=128 nt)	Time/Seq (=512 nt)	Device
CycleFold [54]	4.117 s	57.927 s	CPU
ProbKnot [15]	0.261 s	4.332 s	CPU
LinearPartition [53]	0.103 s	0.602 s	CPU
CONTRAfold [45]	0.043 s	0.531 s	CPU
LinearFold [46]	0.087 s	0.306 s	CPU
RNAfold [12]	0.024 s	0.166 s	CPU
MXfold2 [31]	0.204 s	3.117 s	GPU
SPOT-RNA [26]	0.847 s	1.877 s	GPU
SincFold [52]	0.267 s	2.661 s	GPU
DSRNAFold [32]	0.056 s	0.756 s	GPU
UFold [28]	0.041 s	0.346 s	GPU
Ours	0.032 s	0.154 s	GPU

## Data Availability

The data were derived from the following resources available in the public domain: RNAStrAlign at https://huggingface.co/datasets/multimolecule/rnastralign (accessed on 15 January 2026); ArchiveII at https://huggingface.co/datasets/multimolecule/archiveii.1024 (accessed on 15 January 2026); bpRNA-1m at https://huggingface.co/datasets/multimolecule/bprna (accessed on 15 January 2026).
